# Cost-Effectiveness Analysis of Integrated Bite Case Management and Sustained Dog Vaccination for Rabies Control

**DOI:** 10.4269/ajtmh.22-0308

**Published:** 2023-05-15

**Authors:** Emma Taylor, Joaquin M. Prada, Victor Del Rio Vilas, Eduardo A. Undurraga, Ryan Wallace, Daniel L. Horton

**Affiliations:** ^1^ School of Veterinary Medicine, University of Surrey, Guildford, United Kingdom;; ^2^ Escuela de Gobierno, Pontificia Universidad Católica de Chile, Santiago, Chile;; ^3^ Research Center for Integrated Disaster Risk Management (CIGIDEN), Santiago, Chile;; ^4^ CIFAR Azrieli Global Scholars Program, CIFAR, Toronto, Ontario, Canada;; ^5^ National Center for Emerging and Zoonotic Infectious Diseases, Centers for Disease Control and Prevention, Atlanta, Georgia

## Abstract

The successful prevention, control, and elimination of dog-mediated rabies is challenging due to insufficient resource availability and inadequate placement. An integrated dog bite case management (IBCM) system plus dog vaccination can help address these challenges. Based on data from the IBCM system in Haiti, we conducted a cost-effectiveness evaluation of a newly established IBCM system plus sustained vaccination and compared it with 1) a no bite-case management (NBCM) and 2) a non–risk-based (NRB) program, where bite victims presenting at a health clinic would receive post-exposure prophylaxis regardless of risk assessment. We also provide cost-effectiveness guidance for an ongoing IBCM system and for sub-optimal dog vaccination coverages, considering that not all cost-effective interventions are affordable. Cost-effectiveness outcomes included average cost per human death averted (USD/death averted) and per life-year gained (LYG). The analysis used a governmental perspective. Considering a sustained 5-year implementation with 70% dog vaccination coverage, IBCM had a lower average cost per death averted (IBCM: $7,528, NBCM: $7,797, NRB: $15,244) and cost per LYG (IBCM: $152, NBCM: $158, NRB: $308) than NBCM and NRB programs. As sensitivity analysis, we estimated cost-effectiveness for alternative scenarios with lower dog-vaccination coverages (30%, 55%) and lower implementation costs. Our results suggest that better health and cost-effectiveness outcomes are achieved with the continued implementation of an IBCM program ($118 per life-year saved) compared with a newly established IBCM program ($152 per life-year saved). Our results suggest that IBCM is more cost-effective than non-integrated programs to eliminate dog-mediated human rabies.

## INTRODUCTION

Rabies is a neglected zoonotic disease with the highest case-fatality rate of any known infectious disease. Without the prompt administration of post-exposure prophylaxis (PEP), once clinical signs develop, rabies lyssavirus infection will result in a progressive, fatal encephalitis.^1^^,^^2^ Despite the availability of an efficacious vaccine for those potentially exposed to rabies, dog bite victims may not seek medical advice due to cost and limited health care access. Pre-exposure prophylaxis is also available for those deemed at high risk of rabies virus exposure and consists of a series of vaccinations administered prior to potential exposure. However, similar resource allocation limitations, stock availability, costs, and travel to health facilities mean that those most vulnerable are often not receiving vaccinations.^3^^,^^4^

Most human rabies cases are caused by transmission from domestic dogs. The prevention of human rabies depends upon the effective and verifiable control of the disease within the domestic dog population.^5^^,^^6^ Dog vaccination is available as part of routine veterinary services or mass vaccination campaigns conducted in regions where the disease still circulates among the canine population.^7^ Eliminating dog-mediated rabies is fundamental to meeting the goal of zero dog-mediated human rabies by 2030.^8^

Despite some regional successes in rabies control, rabies remains a disease of global concern, affecting approximately 150 countries and territories.^9^^,^^10^ In Haiti, on the island of Hispaniola, the burden of rabies continues despite attempts at control. Located in the Greater Antilles archipelago of the Caribbean, Haiti has an estimated population density of 414 people per km^2^.^11^ Haiti is the poorest country in the Western Hemisphere.^12^ Identifying cost-effective methods for rabies control in such settings is crucial, given the limited funding and resources available and the many competing health priorities.

Budget constraints result in reduced passive surveillance, insufficiently trained professionals, and reduced availability of PEP.^13^^,^^14^ A dog population survey in 2014 estimated the dog population in Haiti likely to be over one million.^15^ In 2015, historical vaccination records were updated to reflect the dog population survey results and showed that dog vaccination coverage was probably as low as 15% between 2010 and 2012. Procurement of vaccines based on this new dog population figure increased vaccination coverage to approximately 55% in 2015.^13^^,^^15^

In 2013, the CDC collaborated with the Haitian government to initiate the Haiti Animal Rabies Surveillance Program (HARSP), an integrated bite case management (IBCM) system that combines active community-based dog bite investigations with passive animal rabies investigations. The active component of the community bite investigation is conducted via active surveillance and sampling of found dead dogs, coupled with counseling of bite victims. The passive component of the investigation is dependent on reports generated from medical centers and community members. However, the program requires extensive support, with a large team trained in rabies surveillance needed for fieldwork. These control officers are responsible for locating suspected rabid animals involved in bite events and completing a rabies assessment. During assessment of the biting dog, all clinical signs, vaccination status, sex, and estimated age are recorded. If successful capture of the dog occurs, it is either euthanized immediately and tests submitted for diagnosis, or the animal is placed in quarantine in the owner’s home and observed for 14 days. The CDC recognizes six components of HARSP: surveillance, laboratory capacity, public education, responsible dog ownership, mass dog vaccination campaigns, and government engagement. When combined, these components increase dog rabies case detection and can prevent transmission to humans.^16^ Dog bite investigations allow for the removal of rabid dogs from the community, reducing the risk of potential exposures and encouraging bite victims to seek appropriate medical care. Estimates suggest that the risk of being bitten by a rabid dog is relatively low in Haiti (∼0.3%),^9^ based on the relatively small proportion of dogs that test positive. Hampson et al.^9^ produced regional model estimates of biting incidence per 100,000 and reported 238.6 for Haiti. Schildecker et al.^17^ received over 2,000 survey responses to assess dog ecology and barriers to rabies control in dog populations in Haiti. The study estimated a bite incidence of 0.32 per 100,000 population across 14 communes in four departments. This suggests that there are large differences between field-derived and modeled estimates of bite rates.

Dog vaccination campaigns have been widely reported as an effective prevention and control strategy.^9^^,^^13^^,^^18^^–^^23^ A study by Undurraga et al.^24^ suggests that HARSP was cost-effective compared with the baseline scenario of treating dog bite victims who report to the health system as suspected rabies exposures.^24^ Coupling an IBCM with dog vaccination campaigns may be especially efficient in countries where the risk of dog-mediated rabies transmission is high and dog vaccination coverage is challenging to manage.

In this study, we aim to guide decision-makers and policymakers in regions where no IBCM system exists by estimating the cost-effectiveness of introducing an IBCM system into a new country or region beyond Haiti, including surveillance, training, and diagnostics, and including alternative dog vaccination coverage rates for 5 years. We also provide cost-effectiveness estimates for an ongoing IBCM system and sub-optimal dog vaccination coverages, considering that not all cost-effective interventions are affordable. To do this, we considered two scenarios: one where an IBCM system is a new implementation with a high up-front cost (scenario 1) and one where an IBCM system is already in place, so only costs for maintaining the program are needed (scenario 2). In both scenarios, we considered a range of dog vaccination coverage achieved (30%, 55%, and 70%) and compared the costs and health outcomes of IBCM against no bite-case management (NBCM) and a non–risk-based (NRB) program over 5 years.

## MATERIALS AND METHODS

### Data sources.

Historical records detailing bite reports from dogs for 2016 were made available from Ministere de la Sante Publique et de la Population and Ministere de l’Agriculture des Ressources Naturelles et du Développement Rural from 7 of the 10 departments across Haiti (see Supplemental Materials 1). These data were used to inform the epidemiological characteristics in our simulations. They included total human rabies exposures and type of exposure (as defined by WHO guidelines). The categories of health status in dogs (i.e., confirmed, probable, suspected, and negative) are only known because an IBCM system is in place.

The cost data were divided into capital costs (vehicles, animal capture equipment, communication devices), operational costs (vehicle maintenance, fuel for vehicles, office rental, and utilities), and personnel costs (salaries without fringe benefits). Historically, as part of ongoing efforts to control dog rabies via the HARSP program, most dog vaccines have been donated. Therefore, the cost of the individual dog vaccines was not included in this analysis. The cost of vehicles was estimated based on the cost of one rental vehicle per day and multiplied by the length of time in use. Diagnostic costs were also divided into capital costs (fluorescent microscope, incubator, solar freezers, and biological safety cabinet), operational costs (equipment maintenance, insurance, office rental and utilities, personal protective equipment, and reagents), and personnel costs (laboratory technicians, salary, personal protective equipment). Training costs included operational costs (field and classroom days, cost per day of each participant, training supplies), and personnel (teacher, salary, travel expenses). Training costs are assumed to occur each year. PEP costs are based on a cost per dose ($14.45/dose), including the cost of administration (needles, swabs, cold-chain storage), cost per outpatient visit, and overhead cost per visit.

**Figure 1.**
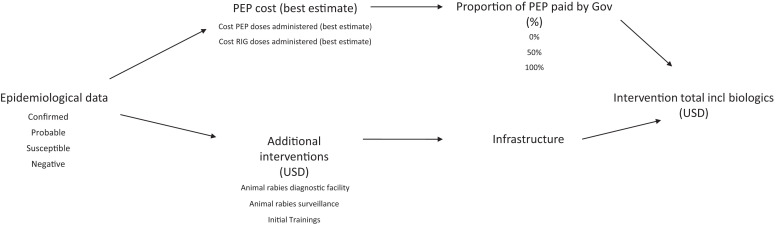
Flowchart detailing inputs needed for determining the total cost of intervention, including the cost of biologics. Epidemiological data (bite victims’ data) supplied from Haiti Animal Rabies Surveillance Program determines the costs of post-exposure prophylaxis (PEP) and rabies immunoglobulin (RIG) needed. Intervention costs determine those costs attached to implementing an integrated bite case management system in regions epidemiologically similar to Haiti. They include capital, operational, and personnel costs attached to surveillance, diagnostic, and training components of the program. The proportion of PEP paid by government is set at either 0%, 50%, or 100%.

Costs (training, communication, vaccination campaign equipment, coordination) were supplied by the CDC based on their collaboration with the government of Haiti, expressed as 2016 US dollars (USD). Costs from Haiti were used as a baseline for the simulations (gross domestic product [GDP] per capita $870 for 2016).^12^ The costs of PEP materials were taken from published literature, and all other costs detailed above (surveillance, diagnostics, and personnel) were kept as the annual costs detailed by Undurraga et al.,^24^ also provided by the CDC. Conservative estimations were given for years of useful life for the equipment. Annual capital costs (USD) estimated show the equivalent annual cost for the capital outlay, where useful life years for the equipment was either 5 or 10 years and where it is assumed that the resale value is zero. Costs, excluding those associated with training, were estimated using constant dollars (with no inflation) and used a time discount rate of a recommended 3%.^25^^–^^28^ The costs considered in the analysis are summarized in Figure 1.

### Scenarios for cost-effectiveness simulations.

We conducted a cost-effectiveness analysis for different scenarios over a 5-year period, assuming a PEP compliance in bite victims of 54% achieved in a human population of around 9.5 million humans and 800,000 dogs (using data from seven departments across Haiti as a reference). We assumed that 54% of bite victims would seek medical care based on data collected in 2014–2015 in a study in the West Department, Haiti.^24^ We test this assumption with a sensitivity analysis. We considered three programs for rabies control: 1) a NBCM program, where PEP is offered to bite victims who report to a health facility but where no passive surveillance or community investigation happens; 2) an IBCM system; and 3) a NRB program, where bite victims who present at a health clinic will receive PEP regardless of the result of an exposure risk assessment. These programs have active and passive surveillance components (Table 1).

**Table 1:** The three approaches assessed to complete cost-effectiveness analysis

Intervention	Post-exposure prophylaxis	Public education	Dog vaccination	Passive surveillance	Active surveillance			
					Community investigation	Risk-based assessment	Contact tracing	Trained health workers
NBCM	√	X	√	√	X	X	X	X
*IBCM, ^Ω^IBCM	√	√	√	√	√	√	√	√
NRB	√	X	√	√	√	X	X	X

ICBM = integrated bite case management; *IBCM = ICBM where the assumption is that 6% of bite victims do not seek treatment despite advice from Haiti Animal Rabies Surveillance Program (HARSP) (scenario 1); ^Ω^IBCM = ICBM where the assumption is that 100% of bite victims seek health care after HARSP advice and capital costs for surveillance and diagnostic components of IBCM are removed (scenario 2); NBCM = no bite-case management; NRB = non–risk-based approach where bite victims who present at a health clinic will receive post-exposure prophylaxis regardless of the result of an exposure risk assessment.

With NBCM as the reference (status quo) program, we explored two scenarios for an IBCM system and a NRB program: a new implementation of the program with high upfront costs (scenario 1) and maintenance of the program, where only costs of running the ongoing program are considered (scenario 2). In scenario 1, as a new implementation of an IBCM system, we assumed that 6% of patients would not seek treatment despite the availability (*IBCM).^24^^,^^29^ The estimate of the share of people who did not seek medical care following an animal bite (6%), despite IBCM counseling advice, was obtained from published literature.^29^ In contrast, in scenario 2, we assumed all patients would seek treatment (^Ω^IBCM). Further, under NRB intervention, 100% of bite victims receive PEP and rabies immunoglobulin treatment regardless of whether the biting dog is confirmed rabid or not. Even bite victims from non-rabid dogs are treated as suspected exposures and therefore receive treatment.

Across both scenarios we considered 30%, 55%, and 70% dog vaccination coverage achieved. The three dog vaccination coverages considered were based on previous findings for coverage achieved in Haiti^19^^,^^30^ and WHO’s recommended 70%.^21^^,^^31^^,^^32^ The probability that a dog that bites had rabies was estimated at 0.063 (baseline value: 6.3%, lower bound: 1%, upper bound: 36%), obtained from estimates for Haiti,^9^ and is assumed to be constant throughout the 5 years for 30% vaccination coverage. When achieving a coverage of 55% or 70%, the probability that the offending dog was rabid is reduced from 6.3% to 1%. The probability of developing rabies if bitten by a rabid dog and without PEP is 19%, again taken from published literature (Equation 1).^33^ An ideal life expectancy, taken from Global Burden of Disease 2010, was used to avoid attributing higher weights to deaths in richer communities.^34^

$$\begin{array}{c} \text{Probability}\;\text{that}\;\text{offending}\;\text{dog}\;\text{is}\;\text{rabid}=\\ \frac{\left(\text{number}\;\text{of}\;\text{dogs}\;\text{confirmed}\;\text{rabid}+\text{number}\;\text{of}\;\text{probable}\;\text{rabid}\;\text{dogs}\right)}{\text{total}\;\text{number}\;\text{of}\;\text{investigations}} \end{array}$$ (Eq. 1)

The analysis was completed from the perspective of the national government. WHO-CHOICE (Choosing Interventions that are Cost-Effective) defines a program as cost-effective if it costs up to three times or less the national annual GDP per capita per disability-adjusted life-year (DALY) averted. One DALY represents the loss of an equivalent 1 year of complete health. In the case of rabies, which is 100% fatal and does not cause disability, one DALY is equivalent to 1 year of life. WHO-CHOICE also defines an intervention as highly cost-effective if it costs up to one time the national annual GDP per capita per DALY averted. This study uses highly effective definition from WHO-CHOICE, the most restrictive threshold as a willingness-to-pay indicator.^30^^,^^35^ More details on the methodology are available in the Supplemental Materials.

### Cost and effectiveness indicators.

The cost-effectiveness for each intervention was estimated as the average cost (USD) per human rabies death averted and the average cost per life-year gained (LYG). Estimated human rabies infections were calculated from the proportion of people bitten (categorized as confirmed, probable, or suspected), the probability that the offending dog was rabid,^32^ and the probability of acquiring rabies if exposed with no PEP.^18^ For the more conservative assumption regarding the dog vaccination coverage achieved (only 30%), rabies prevalence was assumed not to decrease. The probability that a biting dog is rabid is kept at 6.3% throughout the 5-year intervention. When achieving a coverage of 55% or 70%, the probability that the offending dog was rabid is reduced from 6.3% to 1%. This study assumed that bite victims who sought medical care and received PEP did not develop rabies regardless of the reported overall compliance (i.e., completing the five-dose course). A complete list of the variables, their description, and values applied to the cost-effectiveness model are detailed in Supplemental Table 2.

To summarize the cost-effectiveness of all interventions evaluated, the incremental cost-effectiveness ratio (ICER) is used, which can be calculated as follows:

$$\text{Incremental}\;\text{cost}-\text{effectiveness}=\frac{{\text{Cost}}_{1}-{\text{Cost}}_{0}}{{\text{Effectiveness}}_{1}-{\text{Effectiveness}}_{0}}$$ (Eq. 2)

### Sensitivity analysis.

Published data were used to inform a sensitivity analysis. HARSP data, as published by Undurraga et al.,^24^ found that 54% of bite victims presented at health facilities for treatment. A 2013 study estimated that 34% of bite victims presented at health facilities, with 31% initiating rabies vaccination.^30^^,^^36^ In contrast, 80% of bite victims seek medical care, with less than 65% initiating rabies vaccination in Tanzania.^37^ We conducted a sensitivity analysis for scenarios 1 and 2 to observe the impact on average cost per human rabies death averted and the average cost per LYG, using the following variables: 1) share of PEP regimes paid by the government and 2) probability that bite victims would seek medical care and receive PEP. Uncertainty was accounted for by adjusting the share of PEP paid by the national government (lower bound 0%, expected value 50%, and upper bound 100%), and adjusting the probability that a patient would seek medical care (54% IBCM baseline data, and upper 85% and lower 15% bounds).

A full list of assumptions is available in the Supplemental Materials.

## RESULTS

### Cost and effectiveness indicators.

We compared IBCM and NRB systems with an equivalent counterfactual intervention (NBCM) for both scenarios.

**Figure 2.**
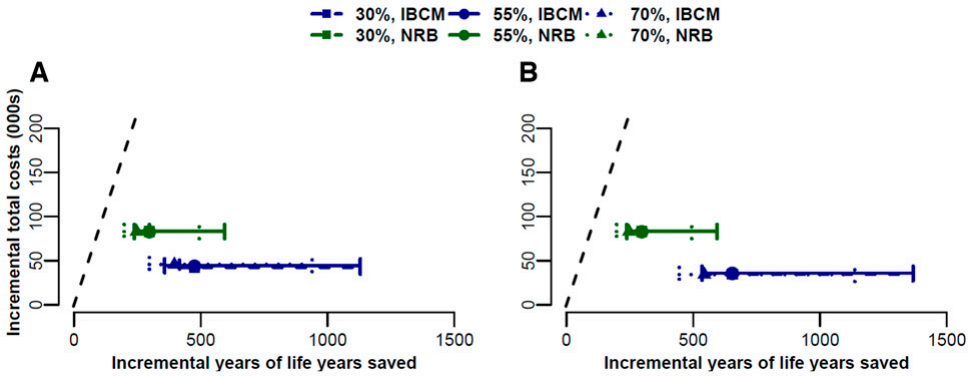
(**A**) Scenario 1. A cost-effectiveness evaluation for setting up an integrated bite case management (ICBM) system, where we assumed that 6% of patients would not seek treatment despite the availability, from scratch for a new country or region with no rabies control program, using limited laboratory and diagnostic capabilities (no bite-case management) as a reference, and compared with a non–risk-based (NRB) approach. (**B**) Scenario 2. A cost-effectiveness evaluation for continuing the established ICBM system we assumed all patients would seek treatment, where capital costs are not considered. The additional vaccination coverages components of (a) 30%, (b) 55%, and (c) 70% were evaluated for the effect on cost across both scenarios and based on the estimated 800,000 dog population across the seven departments, where the probability of developing rabies if bitten by a rabid dog and in the absence of post-exposure prophylaxis is 19%, and the probability that the offending dog had rabies is 6.3%. The diagonal dashed line shows the maximum acceptable incremental cost-effectiveness ratio (ICER). Both the IBCM and NRB interventions fall below the maximum acceptable ICER and can be deemed cost-effective. Solid green and blue lines illustrate the range of uncertainty in the prevalence of disease, which changes incremental life-years saved but does not affect the incremental total cost (000s).

**Figure 3.**
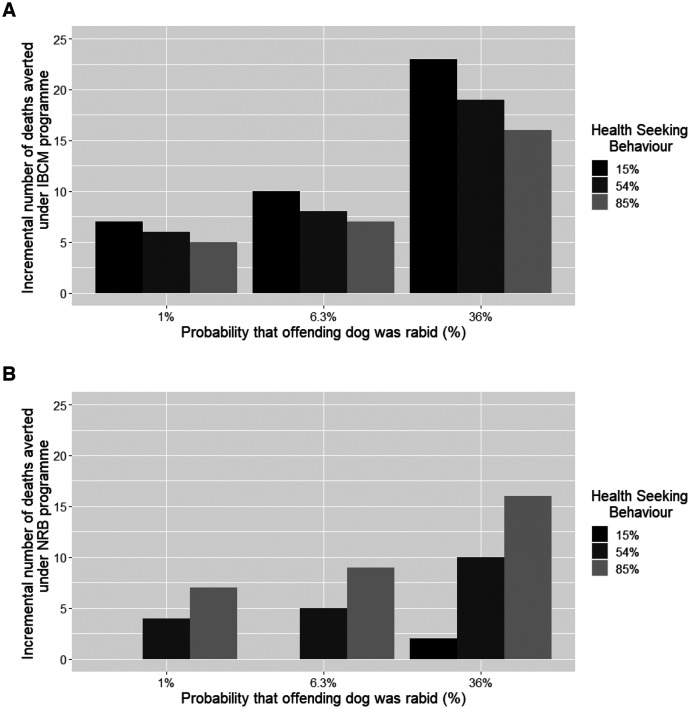
(Scenario 1) Sensitivity analysis for the new implementation of an integrated bite case management (IBCM) system by varying the proportion of bite victims who seek medical care after an incident out of the total number of patients who reported to the IBCM (lower bound: 15%, baseline: 54%, upper bound: 85%) and varying the probability that the offending dog was rabid (lower bound: 1%, baseline: 6.3%, upper bound: 36%) to assess the impact on the incremental number of deaths averted (USD/death). (**A**) ICBM. (**B**) Non–risk-based (NRB) approach.

Integrated bite case management and NRB cost less than the GDP per life-year saved across both scenarios, but the cost-effectiveness of an IBCM system is generally superior to the NRB program, providing better health outcomes at a lower cost in both scenarios across the vaccination coverages and the probabilities that the offending dog had rabies. Integrated bite case management and NRB interventions fall below the maximum acceptable ICER and can be deemed cost-effective (Figure 2). We illustrate the effect of uncertainty in disease prevalence, which changes the number of life-years saved but does not affect the total cost (Figure 3).

#### Scenario 1, new implementation of an IBCM program.

For scenario 1, the new implementation of an IBCM system, in a situation where a 70% vaccination coverage is achieved, the average cost per LYG was higher under NBCM and NRB programs when compared with the *IBCM system (*IBCM: $152, NBCM: $158, NRB: $308 per year) (Supplemental Table 2). Similarly, the NBCM and NRB programs have higher average costs per death averted when compared with the *IBCM system (*IBCM: $7,528; NBCM: $7,797; NRB: $15,244 per year) (Supplemental Table 2).

#### Scenario 2, maintenance of an IBCM program.

For scenario 2, the continued implementation of IBCM, the average cost per LYG under a NBCM program was higher when compared with ^Ω^IBCM (NBCM: $158, ^Ω^IBCM: $118 per year). Additionally, the cost per death averted was higher under NBCM when compared with ^Ω^IBCM (NBCM: $7,797, ^Ω^IBCM: $5,827 per year) (Supplemental Table 4). Similarly, the average cost per LYG was higher under a NRB program when compared with ^Ω^IBCM (NRB: $308, ^Ω^IBCM: $118 per year). Under a 5-year intervention, the average cost per death averted was higher under a NRB program when compared with ^Ω^IBCM (NRB: $15,244, ^Ω^IBCM: $5,827 per year).

#### Comparison of scenario 1 and scenario 2.

When comparing scenario 1 *IBCM to a fully adhered to ^Ω^IBCM seen in scenario 2, the average cost per LYG was lower in scenario 2 under ^Ω^IBCM (*IBCM: $152, ^Ω^IBCM: $118 per year). Additionally, scenario 2 provides better health outcomes. The average cost per human death averted was also lower in an established program (scenario 2, ^Ω^IBCM) compared with scenario 1 *IBCM (*IBCM: $7,528, ^Ω^IBCM: $5,827 per year).

### Sensitivity analysis.

#### Scenario 1.

We also explored the impact on the cost of 30%, 55%, and 70% vaccination coverage when coupled with *IBCM and compared between two alternative interventions (NRB and NBCM). Our results suggest that a 30% vaccination coverage incurs a lower average cost per LYG than either 55% or 70% vaccination compared with the NBCM program (Supplemental Table 2). When varying the proportion of patients who seek medical treatment (15%, 54%, 85%), we found little variation between the *IBCM 30% vaccination program and the55% vaccination program. For example, when conducting a multivariate sensitivity analysis of the cost per death averted (USD/death), the average cost per death averted for a 30% vaccination program under *IBCM ranged from $3,438 to $7,836 per death averted when varying the share of PEP paid by the government (0%–100%), compared with $4,562–10,275 under a 55% vaccination program under *IBCM (Supplemental Table 5). Additionally, our results suggest that if more health-seeking behavior occurs as a result of the educational and active bite investigation component of IBCM and therefore more bite victims receive PEP (simulated here at values of 15%, 54%, and 85% of bite victims), the overall gains generated from the other components of IBCM become fewer. The more bite victims who benefit from the educational component of the IBCM program or are already aware of rabies and prepared to seek PEP, the less incremental additional benefit of the program. This suggests that IBCM would be most cost-effective in areas where health-seeking behavior is currently the lowest (Supplemental Table 5, Figure 3, Supplemental Figure 1).

Achieving a high vaccination coverage translates to a higher requirement for individuals in the vaccination brigades. For a program reaching around 800,000 dogs, the number of individuals is estimated to be 145 for 30%, 207 for 55%, and 297 for 70% coverage achieved. This increase in operational and personnel costs required for training translated to an estimated total of $1,243 per year, $1,421 per year, and $1,680 per year for vaccination coverages of 30%, 55%, and 70%, respectively. These estimated costs indicate that the costs per percentage vaccination coverage increases with the number of vaccinators increases with vaccination coverage. Lastly, we assumed that the share of PEP paid by the government was 50%, but the estimated net monetary benefit, total economic cost, average cost per human death averted, and average cost per LYG are robust to changes in the share of PEP paid by the government (Supplemental Table 3).

#### Scenario 2

We investigated the cost-effectiveness of continuing an IBCM program (scenario 2). Based on a stable 70% and 55% vaccination coverage and assuming a lower rabies prevalence, we compared the results with a more conservative program of 30% vaccination coverage and assumed rabies incidence does not decrease. Under ^Ω^IBCM, 100% of bite victims receive PEP; therefore, zero years of life lost occur, regardless of vaccination coverage achieved. Because the majority of total costs are due to PEP provision, the impact on overall cost as we increase dog vaccination is small. Further, although the total cost of intervention will increase, we see the impact occurring on cost-effectiveness, with the average cost per death averted increasing from $4,730 for a 30% coverage to $5,805 for a 55% coverage and $5,827 for a 70% coverage under ^Ω^IBCM. Additionally, the average cost per LYG increases from $80 for a 30% coverage to $98 for a 55% coverage and to $118 for a 70% coverage (Supplemental Table 4).

## DISCUSSION

Overall, the results from our cost-effectiveness analysis suggest that implementing an IBCM program is more cost-effective than a program without case management or risk assessment. As per WHO guidelines, vaccinating 70% of dogs over 5 years should lead to rabies control and eventual elimination.^31^ However, 70% coverage is not always achieved, particularly early in control programs; additionally, it may take a considerable number of years to reach 70% coverage, during which time many people may have died of rabies. Our results suggest that an IBCM system is still cost-effective while dog vaccination programs are developing and have not yet reached the required 70% to eliminate rabies. Based on data from Haiti, these results can help inform other regions to support the implementation of an IBCM system. However, local costs are required to inform the local economic burden accurately.

Alternative vaccination coverages were explored. With the proportion of bite victims seeking treatment at 54% and a 6.3% probability that the offending dog is rabid, a 30% vaccination strategy yields a reduction in the cost per death averted compared with a 55% vaccination program. A low vaccination coverage may still provide marginally more cost-effective results for a region looking to implement an IBCM-style system but is unlikely to achieve rabies elimination unless a higher vaccine coverage can be achieved. This apparent marginal improvement in cost-effectiveness is misleading because a lower coverage is unlikely to achieve rabies elimination and therefore cannot be recommended. Such a program would increase costs to the government when converting from NBCM to an IBCM-style program, mainly due to the animal surveillance and staff training components, which vary depending on program needs. It is important to note that these analyses only assess the economic impacts of these different interventions and cannot be used to justify a 30% vaccination coverage alone. That is, the cost-effectiveness of low-coverage programs, such as the 30% coverage scenario explored in this study, worsens as you extend the period of evaluation. Although the initial cost-effectiveness evaluations under the time period analyzed in this study may yield acceptable results, longer economic periods of analysis would likely show that implementing a lower vaccination coverage is an unwise approach. Further work exploring the impact of lower vaccination coverage on eliminating rabies but including ancillary benefits of an IBCM, such as responsible dog ownership, education and outreach, and public engagement, would be important and is outside the scope of this study. The NBCM and NRB programs do not contain active bite case management in their animal surveillance component (Table 1). Despite adding costs due to staff training, completing targeted risk assessments of all bite victims in an IBCM leads to improved PEP compliance.

Additionally, the share of bite victims who seek medical care due to active bite investigations was estimated at 40% in Haiti’s HARSP.^38^ Moreover, as countries take ownership of the program, the cost of PEP might shift from donors to national governments, changing the cost-effectiveness. In 2013, an estimated 20,000 Vero cell rabies vaccines and later, in 2017, a further 15,000 rabies vaccines were donated to Haiti by Brazil.^28^ However, a rabies control program based solely on PEP donations is unlikely to be sustainable or effective.^14^ It is unknown how long a national government or donor would subsidize PEP, which needs to be considered.^24^ That being said, PAHO’s Revolving Fund for Access to Vaccines contributes to the sustainability of National Immunization Programs by providing affordable, high-quality vaccines to countries in the Western Hemisphere.^39^ Additionally, the inclusion of low-cost intradermal vaccines adoption may affect these findings further.^40^^,^^41^

The impact of low-level dog vaccination in reducing rabies transmission below the recommended 70% coverage is not well understood.^24^^,^^42^ There is little published evidence detailing what, if any, vaccination coverage needs to be established in regions that have reached a canine rabies–free status. Without this valuable knowledge, these areas are at risk of reverting to endemic regions with the reintroduction of the virus. A study by Jeon et al.^8^ concludes that a vaccination coverage of 38–56% throughout susceptible dog populations may be needed to maintain a rabies-free status and mitigate the risk of reintroduction. Further, vaccine coverage needed to sustain a rabies-free status varies substantially by region and depends on dog–dog transmission rates and frequency or risk of re-introduction.^42^ Understanding how dog movement affects rabies elimination timelines under different vaccination regimes is key to designing appropriate interventions and achieving the ambitious 2030 goal of zero dog-mediated human rabies deaths.^43^

All known program costs have been considered in these analyses, but some aspects are locally specific. Quarantine costs will vary from country to country and need to be incorporated into planning. In our study, dogs put under observation incurred no additional cost to the government because quarantines are performed on the owner’s property and are thus not included in the results.

Our assumptions regarding the probability that the biting dog was rabid are taken from published estimates for Haiti. However, these will differ from region to region, as will dog-keeping practices and dog behaviors, which will affect these estimates. Understanding the risk associated with a biting dog may help reduce unnecessary PEP administration and costs if, after a biting incident, an assessment of the risk is deemed sufficiently low. Importantly, establishing a reliable case definition encourages bite victims to seek medical advice and identifies bite victims of non-rabid dogs, thus reducing the waste of resources and helping to improve the program’s cost-effectiveness. These WHO Case Definitions are important for determining the need for PEP administration but are less useful for describing rabies epidemiology in dog populations. The WHO definition states that any dog with an unknown outcome be classified as “probable” for rabies risk, meaning that the risk for transmission after a bite event is high. Conversely, a study by Ma et al.^44^ assesses the quantitative rabies risk in dogs in Haiti and is therefore able to more accurately understand the epidemiological situation and associated risk of rabies in dogs when compared with using the WHO case definition alone. Both the WHO Case Definition and mathematical modeling are necessary for evaluating an intervention strategy but differ greatly in their uses. Case definitions are useful for supporting PEP decisions, whereas modeling assists with epidemiological understanding.

However, achieving zero wastage of PEP to low-risk bite victims is deemed unlikely in dog rabies endemic areas. First, vaccine from vials must be discarded after 6–8 hours of being opened, and rabies exposures make up only a small proportion of dog bites in Haiti.^21^^,^^37^ Within our deterministic model, the rabies infection categories are defined as confirmed, probable, suspected, and negative and used only as a reference. IBCM is the only program in which this information would be known. The total dog investigations conducted annually and the clinical outcomes of those dogs show that the number of confirmed, probable, and suspected rabid dogs can increase when programs are implemented. This may be explained because the community and training staff involved in the intervention become accustomed to reporting and following up on bite victims. However, data on the number of reported bites from dogs from recently dog rabies–free countries suggest that, even when a vaccination intervention is in place, the number of actual dog bites occurring does not change. Although rabies among dog bite victims is very rare, dog bites in general are very common, with rabies rarely being the reason for the bite taking place. This further highlights the importance of completing a risk assessment to ensure that PEP is not administered for low/no risk bites, which will grow in proportion as the control program becomes more successful. Moreover, the number of dogs that test negative for rabies increases yearly as more and more dogs get vaccinated and surveillance improves. Additionally, the number of diagnostic tests completed increases as we move from testing only the most likely rabid cases to diagnosing all rabies-suspected dogs. It would be reasonable to expect a lower proportion of cases being rabid.

The proportion of PEP paid by the government and the probability that the offending dog was rabid have minimal effect on cost-effectiveness in the sensitivity analyses at low vaccination levels (30% and 55%). It was further noted that, under an IBCM, the average cost of human deaths averted is marginally lower under a 30% vaccine coverage program. Under a non–risk-based program, all exposures are treated as suspected rabies exposure, which may account for this variation. An IBCM system conducts active community-based investigations, advising bite victims on a case-by-case situation and promoting a risk-based PEP decision-making process. Therefore, the IBCM system was not greatly affected by varying the proportion of patients who sought treatment and the probability that the offending dog was rabid. There have historically been insufficient data available that capture how, when, and why bite victims access health facilities. However, a study by Etheart et al.,^38^ which assessed the effects of counseling bite victims and the adherence to PEP in Haiti, found that high proportions of bite victims started PEP after completing counseling, with those who had confirmed or probable exposure most likely to complete the five-dose course. This complimentary service provided by IBCM has been shown to increase health-seeking behavior when run alongside mass vaccination campaigns. As such, an IBCM system should not substitute mass vaccination.^38^ This advocates for programs to remain flexible because needs will vary with changes in disease prevalence. The IBCM comprises many different components, such as training brigades, which may not need to be repeated yearly or at later stages of the implementation. Further work is needed to assess which components of the IBCM could be removed later in the implementation cycle.

Our evaluation of the cost-effectiveness of maintaining an IBCM in Haiti (scenario 2) projected its effects over 5 years and, similarly to scenario 1, included a lower dog vaccination rate of 30% compared with the WHO-recommended 70%. The 30% and a 70% interventions both proved to be cost-effective. However, it is unlikely that 30% vaccination will achieve rabies elimination within the region. Further, as a region moves toward rabies elimination, mass vaccination costs remain high, and dog transmission decreases because fewer and fewer rabid dogs remain. Therefore, the number of human deaths averted will decrease until they reach zero. When considering a short-term evaluation, the incremental cost-effectiveness decreases. Mass dog vaccination should have long-term benefits leading not only to the elimination of dog rabies cases but also to a reduction in PEP and laboratory costs and increased surveillance benefits, all of which are included in the IBCM.^21^^,^^30^ For example, a recent study showed that the national rabies control program in Mexico that eliminated dog–human rabies transmission in 2019 has been highly cost-effective by WHO-CHOICE standards.^45^

Our projections for Haiti cannot determine how long it would take an IBCM to reduce the prevalence of rabies in the country, so prevalence was only assumed with each scenario. Additionally, projecting cost and epidemiological changes is difficult. Although deterministic in approach, Borse et al.^42^ show the potential effect of vaccinating different proportions of the dog population and how rabies control may be achieved with a lower than 70% vaccination coverage, depending on existing disease transmission. It is important to use mathematical models calibrated with field data to understand the effect of achieving different dog vaccination levels depending on the local dynamics of dog rabies transmission.

The reduction in the probability of being bitten by a rabid dog will be affected by changes in rabies transmission rates.^42^ Our study includes extensive sensitivity analysis, including varying the proportion of patients who seek medical care, the probability that the offending dog was rabid, and the share of PEP paid by the government. The main results did not change. However, uncertainty around the share of individuals who do not seek medical care after exposure remains. Published literature estimates that 54% of individuals potentially exposed seek medical care; however, this figure could be much lower. Health-seeking behavior will vary from region to region, depending on the presence or absence of rabies educational campaigns, accessibility to health care, associated costs, and other competing diseases of interest. There is also uncertainty regarding the number of bite victims who seek PEP. However, this had little effect on our results because the sensitivity analyses conducted were robust when varying the proportion of PEP paid for by the government and because the number of bite victims seeking PEP was based on real-world data from Haiti. Because the Haitian government provides PEP for free to the population, improving health-seeking behavior and compliance would support the goal of reducing rabies-related deaths. Additionally, our assumptions for the proportion of bite victims not seeking health care (6% and 0% *IBCM and ^Ω^IBCM, respectively) are likely to impact the effectiveness of an IBCM system.

We used WHO-CHOICE thresholds to determine cost-effectiveness. These thresholds seek to assess interventions at the sub-regional level and then classify these interventions into broader groupings to aid decision-makers. However, the application of utility thresholds to aid within-country decision-makers with individual interventions is poorly understood and has been criticized in the literature.^35^^,^^46^ For example, using ICERs to measure cost per unit of health outcome is not intuitive to decision-makers without a reference benchmark of an acceptable value of cost per health outcome. The application of WHO thresholds supports the identification of resource allocation and efficiency of interventions. However, these thresholds do not capture the sustainability of an intervention or the impact of other interventions in place that may have their funding displaced.^35^ All alternative programs for a prevalence of 6.3% are below the WHO-CHOICE threshold and are therefore considered cost-effective. However, interventions using bite-case management are more cost-effective than those without case management or risk assessment.

The results of this study suggest that an IBCM program is a useful and cost-effective public health tool in a range of situations, even after several years of implementation. However, data that reflect the rabies situation locally are needed to make decisions that have a local impact. Such a framework also generates evidence that could be used to inform policy-making and decision-making and support the replication of the IBCM system in other regions.

## Supplemental Material


Supplemental materials
