# Supplementary material for: Cost-Effectiveness Analysis of Integrated Bite Case Management and Sustained Dog Vaccination for Rabies Control
Source: Am J Trop Med Hyg. 2023 May 15;109(1):205–13. doi: 10.4269/ajtmh.22-0308 (PMC10324003; doi:10.4269/ajtmh.22-0308)
Supplement: Supplementary file 1 [file tpmd220308.SD1.pdf]

Supplementary Material

American Journal of Tropical Medicine and Hygiene

Cost-effectiveness analysis of integrated bite case management and sustained dog vaccination for rabies control

*Emma Taylor, Joaquin M. Prada, Victor Del Rio Vilas, Eduardo A Undurraga, Ryan Wallace, Daniel L. Horton

Contents

Table S1. 2
Table S2. 2
Table S3. 3
Table S4. 4
Assumptions 5
Supplementary Figures 7
Figure S1 8

Table S1. (Scenario 1) Annual cost-effectiveness Indicators (average cost (US$) per life year gained and average cost (US$) per human death averted for *IBCM compared to NBCM, and NRB.

	**Average cost (US$) per life year gained**		
	**NBCM**	***IBCM**	**NRB**
30%	74	58	31
55%	131	125	257
70%	158	152	308
	**Average cost (US$) per human death averted**		
	**NBCM**	***IBCM**	**NRB**
30%	6,340	5,637	12,279
55%	7,797	7,419	15,244
70%	7,797	7,528	15,244

Notes. *IBCM where the assumption is that 6% of bite victims do not seek treatment despite advice from HARSP (scenario 1)

Table S2. (Scenario 1) Net monetary benefit as function willingness to pay (WTP) for an additional year of life gained for setting up a *IBCM style programme from scratch for a new region (compared to current situation in Haiti) for 30%, 55% and 70% vaccination scenario. Willingness to pay (WTP) denotes willingness to pay for an additional unit of effectiveness, in this case, effectiveness is measured in years of life saved (YLS). Net monetary benefit of the HARSP programme was calculated as NMB=WTP*Δ effectiveness-Δ cost

NET MONETARY BENEFIT (NMB)				
2016				
SHARE OF PEP BY THE GOVERNMENT	**Willingness to pay**	***IBCM (30%)**	***IBCM (55%)**	***IBCM (70%)**
	Life-year gained	NBM> 0 cost-effective		
	0	-89	-122	-150
50%	500	411	378	350
	870	735	748	720
	1000	911	878	850
	1500	1,411	1,378	1,350

Notes. Willingness to pay for an additional unit of effectiveness. In this case, effectiveness is measured in years of life saved (YLS). Net monetary benefit of the HARSP program was calculated as NMB=WTP*Δ effectiveness-Δ cost

*IBCM where the assumption is that 6% of bite victims do not seek treatment despite advice from HARSP (scenario 1)

Table S3. (Scenario 2) Results of robustness checks for ΩIBCM intervention over five years for the proportion of patients who seek medical care at 54%, and 50% of PEP is paid by the government

	**ΩIBCM (30%)**	**NBCM (30%)**	**NRB (30%)**	**ΩIBCM (55%)**	**NBCM (55%)**	**NRB (55%)**	**ΩIBCM (70%)**	**NBCM (70%)**	**NRB (70%)**
**Average cost per dearth averted (USD/death)**	4,730	6,340	12,279	5,805	7,797	15,244	5,827	7,797	15,244
**Average cost per life year gained (LYG) (USD/LYG)**	80	107	207	98	131	257	118	158	308

Notes. For 30% vaccination coverage the probability that the offending dog was rabid is 6.3%, and the probability of developing rabies in the absence of PEP is 19%. For 55% and 70% vaccination coverage the probability that the offending dog was rabid is reduced from 6.3% to 1%, and probability of developing rabies in the absence of PEP is 19%

ΩIBCM where the assumption that 100% of bite victims seeking health care after HARSP advice, and capital costs for surveillance and diagnostic components of IBCM are removed (scenario 2)

Table S4. (Scenario 1) Range of values when conducting a multivariate sensitivity analysis of the cost per death averted (US$/death), varying the proportion of patients who seek medical treatment after being bitten by a suspected rabid dog, the share of PEP paid by the government (0-100%), and the probability that the offending dog was rabid (1%,6.3% 36) for 30, 55, 70% vaccination coverage of an estimated 800,000 dog population

Average cost US$/human death averted			
**2016 (30%)**	**Probability that the offending dog was rabid 1%**		
**Patients who seek medical treatment**	**NBCM**	***IBCM**	**Non-risk based approach**
**15%**	8,187-10,188	4,467-10,180	8,187-33,789
**54%**	5,414-10,179	4,467-10,180	2,299-28,187
**85%**	4,045-9,648	4,467-10,180	1,460-27,328
	**Probability that the offending dog was rabid 6.3%**		
**15%**	9,800-12,195	3,438-7,836	9,800-40,445
**54%**	4,403-8,277	3,438-7,836	1,852-22,706
**85%**	3,452-8,234	3,438-7,836	1,173-21,958
	**Probability that the offending dog was rabid 36%**		
**15%**	6,052-7,531	1,560-3,554	344-14,216
**545**	2,407-4,526	1,560-3,554	961-11,777
**85%**	1,658-3,955	1,560-3,554	624-11,673
**2016 (55%)**	**Probability that the offending dog was rabid 1%**		
**15%**	8,187-10,190	4,562-10,275	8,187-33,826
**54%**	5,415-10,184	4,562-10,275	2,300-28,224
**85%**	4,045-9,653	4562-10,275	1,460-27,364
	**Probability that the offending dog was rabid 6.3%**		
**15%**	9,800-12,198	3,511-7,909	9,800-40,489
**54%**	4,403-8,281	3,511-7,909	1,852-22,735
**85%**	3,452-8,239	3,511-7,909	1,173-21,987
	**Probability that the offending dog was rabid 36%**		
**15%**	6,052-7,533	1,593-3,588	3,444-14,231
**54%**	2,407-4,528	1,593-3,588	961-11,792
**85%**	1,658-3,957	1,593-3588	624-11,688
**2016 (70%)**	**Probability that the offending dog was rabid 1%**		
**15%**	8,187-10,189	4,671-10,384	8,187-33,790
**54%**	5,415-10,180	4,671-10,384	2,300-28,188
**85%**	4,045-9,648	4,671-10,384	1,460-27,328
	**Probability that the offending dog was rabid 6.3%**		
**15%**	9,800-12,196	3,595-7,993	9,800-40,446
**54%**	4,403-8,277	3,595-7,993	1,852-22,706
**85%**	3,452-8,234	3,595-7,993	1,173-21,958
	**Probability that the offending dog was rabid 36%**		
**15%**	6,052-7,532	1,631-3,626	3,444-14,216
**54%**	2,407-4,526	1,631-3,626	961-11,777
**85%**	1,658-3,955	1,631-3,626	624-11,673

*IBCM where the assumption is that 6% of bite victims do not seek treatment despite advice from HARSP (scenario 1)

Assumptions

Epidemiology Assumptions

i. Puppies born to rabid dogs will not survive and therefore exposed and infected dogs are not included in the birth rate.
ii. Adjustment factors = dog rabies/reported dog rabies = 1
iii. Adjusted bites = reported dog rabies* adjustment factor. Where reported dog rabies included confirmed, probable, and suspected dogs.

Health Seeking Behaviour Assumptions

i. Estimated human rabies infections were calculated from the proportion of people bitten, categorized as confirmed, probable, or suspected, the probability that the offending dog was rabid (Wallace et al., 2015), and the probability of acquiring rabies if exposed with no PEP (Hampson et al., 2009), and the vaccination level being assessed.
ii. To calculate the number of fatal infections, the estimated human rabies infections were combined with the probability that a bite victim sought medical care and received PEP.
iii. This study assumed that those patients who sought medical care and received PEP did not develop rabies, regardless of the reported overall compliance (i.e., completing the five-dose course).
iv. Life expectancy at birth, for males: 63.35 (95%CI: 61.51-65.34) and females 65.31 (95%CI: 63.15-67.52) we assumed half of the population was of each gender.
v. Fatal rabies infection=(share of patients who seek care)*adjusted human rabies infections*(1-% of confirmed, probably, suspect individuals who receive PEP)+(1-(share of patients who seek care + share of patients who seek care with active bite investigation))*adjusted human rabies infections.

Program-Based Assumptions /Cost Effectiveness Assumptions

i. This study uses generalized WHO-Choice (Choosing Interventions that are Cost-Effective) thresholds as a willingness-to-pay indicator.
ii. WHO-CHOICE defines a threshold as three times gross domestic product (GDP) per capita, to identify the cost-effectiveness of interventions. GDP was adjusted to 2018 rate.
iii. Net monetary benefit (NMB) was estimated as a function of WHO-CHOICE for an additional unit of effectiveness, as defined by year of life gained (compared to the situation in Haiti), and calculated as: NMB = WHO-CHOICE * Δ effectiveness – Δ cost
iv. An ideal life expectancy, taken from GBD 2010 was used, to avoid attributing higher weights to deaths in more affluent communities (Murrey et al.,2012).
v. YLLs were calculated by multiplying deaths at each age by the reference standard life expectancy at that age as estimated by BDB 2010 (Murrey et al., 2012).
vi. We assumed that the age distribution of fatal human rabies infections in Haiti is equivalent to that of Tanzania, which has already been characterized (Cleaveland et al. 2002).
vii. Estimations were given for years of useful life for the equipment. Annual capital costs (US$) estimated show the equivalent annual cost for the capital outlay, assuming that the resale value is zero. Costs were estimated using constant dollars (with no inflation) and used a real discount rate of 3% (Drummond et al., 2005).
viii. To complete robustness checks of IBCM, we assumed that all vaccination coverages explored (30, 55, 70%) were achieved and maintained,

ix. To complete a robustness check of IBCM we assumed that as the HARSP continues and vaccination at 30% is achieved year on year over ten years, the probability of being bitten by a rabid dog should reduce as a function of the 30% vaccination coverage, coupled with the counseling component of HARSP.

Accounting for uncertainty

The share of PEP paid for by the government was varied at 0, 50, and 100%, with the probability that patients who seek medical care was kept at 54% (HARSP baseline data), and upper (85%) and lower (15%) bounds applied for comparison.

Uncertainty was accounted for by adjusting the share of PEP paid by the Haitian government, (lower bound 0%, expected value 50%, and upper bound 100%) and health indicators as a function of this uncertainty, assessed.

To complete a robustness check of HARSP, with a reduction in the number of rabid dogs in the population, we assumed that the probability of being bitten by a rabid dog would decrease and in turn so too would the probability of developing rabies as a result.

We assumed that the continued implementation of HARSP should result in fewer rabid dogs diagnosed and more non-rabid dog bites. Our assumption of a lower vaccination rate of 30% will mean that initially, there is a higher probability that fewer dogs will be immune to rabies, when compared to a higher vaccination rate, keeping all other variables constant. We assume that over time a reduction in the probability of the offending dog being rabid will occur and amend the values from 6.3 to 1.3 to reflect this. The probability that the offending dog is rabid is estimated based on empirical data and the aggregate value of all dog investigations available per year, as described by Wallace et al. 2015 and estimated at 6.3%. A reduction to 1.3% probability is computed using an estimate by year: A confirmed exposure = confirmed exposure*number of confirmed in study per year + probable exposure*number probable exposures in stud per year / number of dog investigations/year.

Supplementary Figures

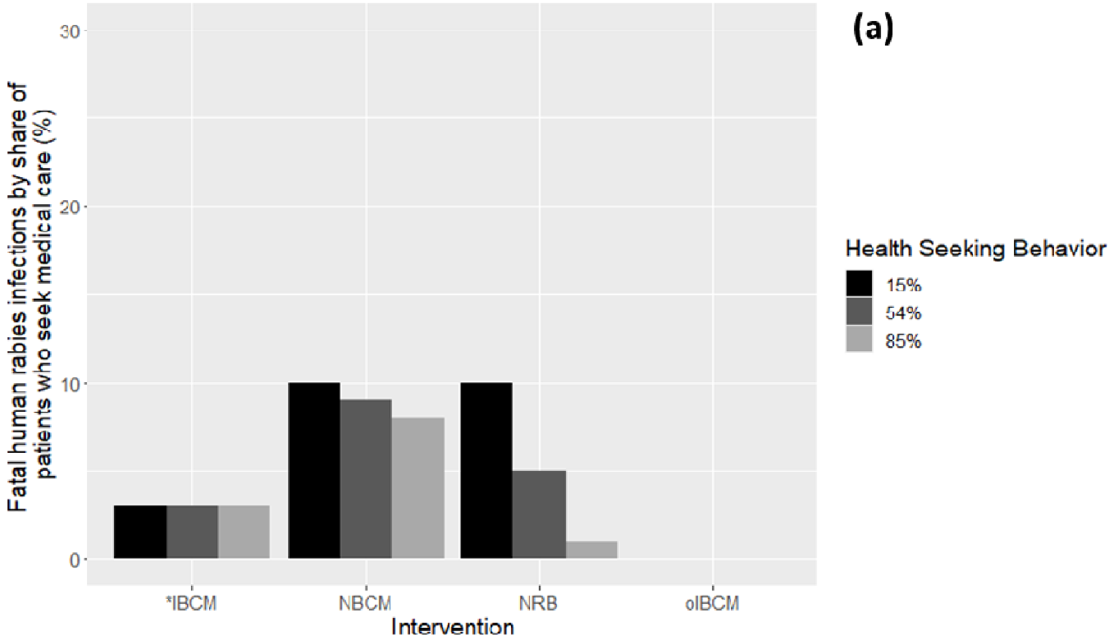

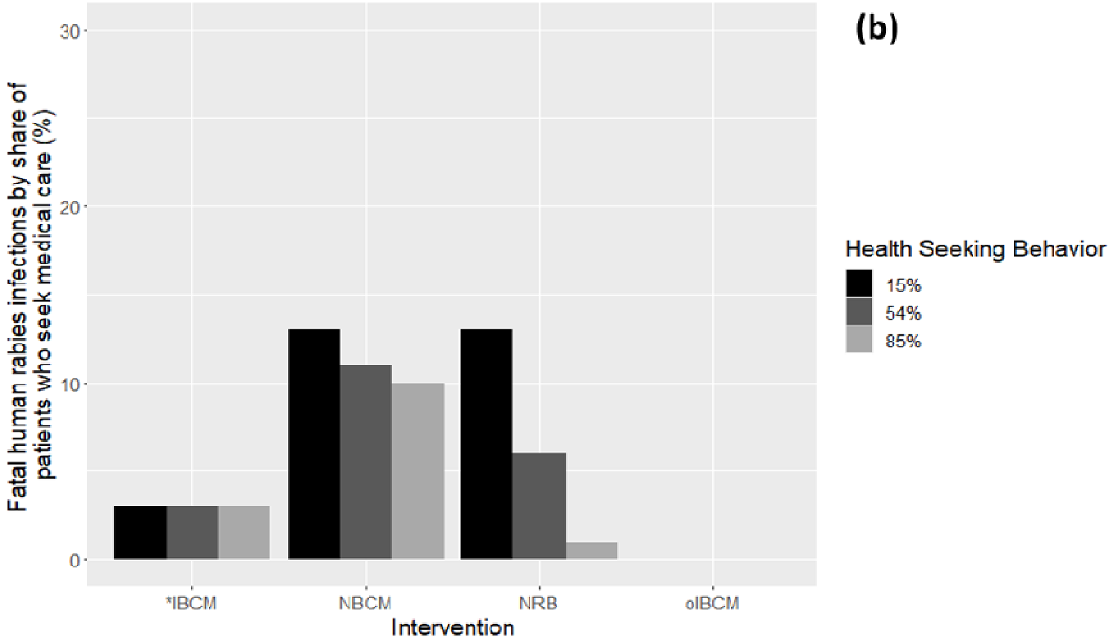

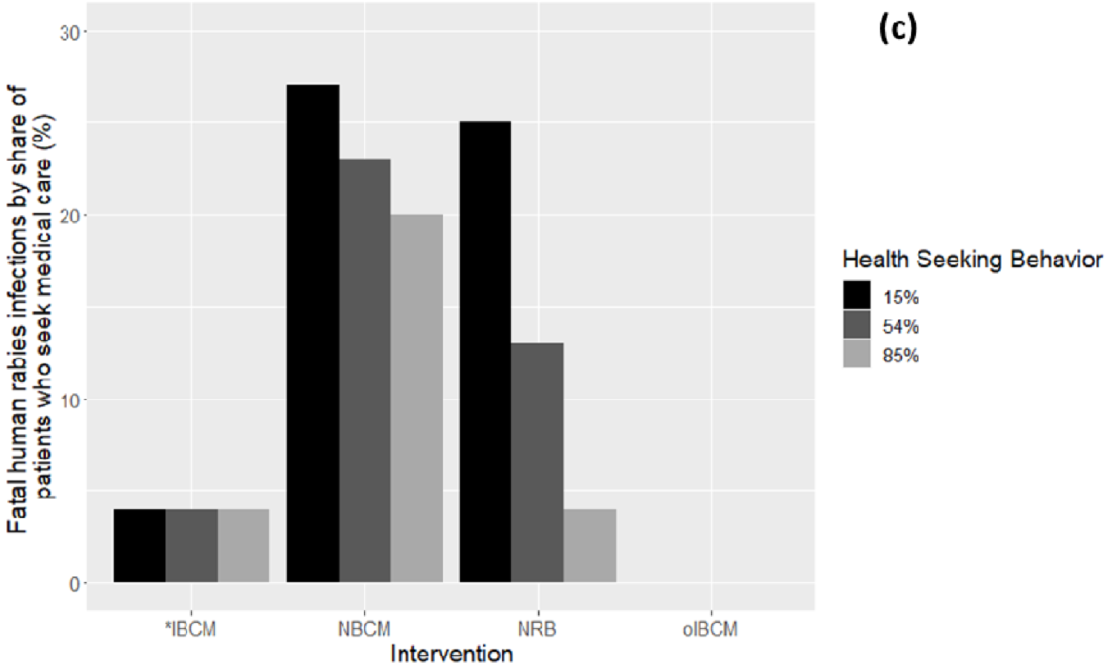

Figure S1. (Scenario 1). Sensitivity analysis for the new implementation of an IBCM, by varying the proportion of patients who seek medical care after a bite incident out of the total number of patients who reported to IBCM (lower bound:15%, baseline: 54%, upper bound: 85%) and varying the probability that the offending dog was rabid (lower bound: 1%, baseline: 6.3%, upper bound: 36%), to assess the impact on fatal number of rabies infections. (a) 1 % probability that offending dog was rabid (b) 6.3% probability that the offending dog was rabid (c) 36% probability that the offending dog was rabid.
